# Bimanual Grasping Adheres to Weber's Law

**DOI:** 10.1177/20416695211054534

**Published:** 2021-11-25

**Authors:** Constanze Hesse, Róisín Elaine Harrison, Martin Giesel, Thomas Schenk

**Affiliations:** School of Psychology, 1019 University of Aberdeen, Aberdeen, UK; Department of Neuropsychology, 9183 Ludwig-Maximilians University, Munich, Germany

**Keywords:** perception–action dissociation, psychophysics, two-visual streams

## Abstract

Weber's law states that our ability to detect changes in stimulus attributes decreases linearly with their magnitude. This principle holds true for many attributes across sensory modalities but appears to be violated in grasping. One explanation for the failure to observe Weber's law in grasping is that its effect is masked by biomechanical constraints of the hand. We tested this hypothesis using a bimanual task that eliminates biomechanical constraints. Participants either grasped differently sized boxes that were comfortably within their arm span (action task) or estimated their width (perceptual task). Within each task, there were two conditions: One where the hands’ start positions remained fixed for all object sizes (meaning the distance between the initial and final hand-positions varied with object size), and one in which the hands’ start positions adapted with object size (such that the distance between the initial and final hand-position remained constant). We observed adherence to Weber's law in bimanual estimation and grasping across both conditions. Our results conflict with a previous study that reported the absence of Weber's law in bimanual grasping. We discuss potential explanations for these divergent findings and encourage further research on whether Weber's law persists when biomechanical constraints are reduced.

## Introduction

The perception–action model (PAM; Milner and Goodale (1995, 2006)) suggests two uses for vision that are independent and governed by anatomically and functionally distinct visual streams in the extrastriate cortex. The ventral stream is assumed to process vision-for-perception, whereas the dorsal stream is assumed to process vision-for-action. The model was originally inspired by studies on patients who suffered from circumscribed damage to either dorsal or ventral stream areas and consequently seemed to show behavioral impairments restricted to either visuomotor or perceptual tasks (e.g., Goodale et al., 1991, 1994). A range of different paradigms has been employed in recent decades to provide evidence for segregated functions also in neurologically healthy humans, with many of them being discussed controversially in the current literature (for reviews see Bruno et al., 2008; Franz & Gegenfurtner, 2008; Schenk & McIntosh, 2010; Smeets & Brenner, 2006). One of the more recent paradigms used to substantiate the claim that vision for action and vision for perception follow different rules also in neurologically intact individuals is the finding by Ganel et al. (2008) that vision for perception but not vision for action adheres to Weber's law.

Weber's law states that humans’ ability to perceive a change in a sensory attribute of a stimulus is inversely proportional to the initial magnitude of this attribute (Fechner et al., 1966). This means that our ability to perceptually discriminate between different stimuli decreases with increasing stimulus magnitude. For example, it is relatively easy to notice a small increase in the size of a small object whereas that same size increment might be imperceptible when applied to a larger object. Adherence to Weber's law is determined by calculating “just noticeable differences” (JNDs; the minimum detectable increment in stimulus magnitude) and examining the relationships between JNDs for different stimulus magnitudes. If the JNDs increase linearly with increasing stimulus magnitude, then they follow Weber's law. This perceptual law has been shown to hold for many different attributes, measurement procedures, and sensory modalities (see Billock & Tsou, 2011; Gescheider, 1997 for reviews).

Surprisingly, however, when Ganel et al. (2008) tested Weber's law in visually guided actions they found that this rule does not seem to hold for human grasping movements. In their study, Ganel et al. (2008) asked participants to either grasp (action task) or estimate the sizes (perception task) of rectangular objects varying in length using their index finger and thumb. They found that while JNDs (measured as the variability in the maximum grip opening) increased linearly with the object size in the perceptual condition, they remained constant across stimulus magnitudes in the grasping condition (for similar results see also, Ganel et al., 2014; Hadad et al., 2012; Holmes et al., 2013; Ozana & Ganel, 2019). This absence of Weber's law in grasping was interpreted as evidence that stimulus size is processed fundamentally differently in perception and action as predicted by the PAM. However, the interpretation of the absence of Weber's law in grasping as empiric evidence for separate visual processing mechanisms for perception and action has since become controversial, too (e.g., Bruno et al., 2016; Holmes et al., 2011; Schenk et al., 2011; Schenk et al., 2017; Smeets & Brenner, 2008).

One important point of criticism that is particularly relevant for our study concerns the role of biomechanical limitations on the grasping response (Utz et al., 2015). Specifically, Utz and colleagues have argued that for larger objects, the hand opening approaches the limits of the absolute hand span, thus reducing the amount of possible variability in the response. Although the maximum grip aperture (MGA) is generally considered a valid measure of the representation of object size in the visuomotor system, other factors—such as sensory noise and biomechanical factors—also affect this measure (Schenk et al., 2017). The larger the object and the closer the required hand opening to the maximum hand extension, the smaller the possible range of safety margin values available. The reduced range of possible openings means that the variability of MGAs (i.e., JNDs) naturally decreases for larger objects, thereby acting against the predictions of Weber's law. The findings that Weber's law seems to hold in grasping for small but not large object sizes (Bruno et al., 2016) and that MGA variability actually seems to *decrease* for larger objects (Utz et al., 2015) support the suggestion that biomechanical limits of the hand may explain the absence of Weber's law in grasping.

However, not all studies seem to be in agreement with this biomechanical explanation. For example, Manzone et al. (2017) demonstrated that Weber's law holds consistently for manual estimation regardless of whether object sizes fall in the small-to-medium range or in the medium-to-large range. This might seem surprising as manual estimation also requires the opening of the hand. Why should biomechanical constraints not also affect the variability in the estimation task? In our opinion, the answer to this question lies in the fact that the potentially constraining effect of the biomechanical limits of the hand is not determined by the size of the object, per se, but by the opening of the hand that is required to successfully perform the object-related task. For manual estimation, the hand-opening generated for a certain object size is, in general, considerably smaller than in grasping. This is because in manual estimation, the hand-opening functions as a non-verbal report of the perceived size. Participants will, therefore, attempt to adjust their hand-opening so that it corresponds as closely as possible to the perceived size of the object. This is not the case in grasping. Here, the hand must open much wider (i.e., beyond the edges of the object) to ensure a good grip on the object. This difference between the MGA and the width of the object is the safety margin. Thus, for the same target object, the hand-opening during grasping will always be closer to the hand's biomechanical limit than during manual estimation. Consequently, the MGA variability will be more constrained in grasping.

There is an additional reason why biomechanical comfort will exert a more pronounced effect on grasping than on manual estimation. The two tasks have very different aims. In grasping, participants are encouraged to perform natural movements, that is, they will aim to pick up objects successfully while also minimizing the effort and the discomfort of the movement (Rosenbaum & Gaydos, 2008). In estimation, however, participants are encouraged to focus on accuracy and are, therefore, more inclined to sacrifice movement efficiency or comfort. Accordingly, grasping will be more susceptible to biomechanical constraints than manual estimation.

Another seemingly challenging finding for the biomechanical account was recently reported by Ayala et al. (2018). In this study, they used object sizes that were adjusted to the size of participants’ hands. That is, an object of size 20% covered 20% of the maximum hand-opening of a specific participant. In the grasping task, they found that MGA variability (i.e., JNDs) remained relatively constant for objects in the range of 20%–80% (of participants’ hand-span) and only decreased for object sizes that exceeded 100% of participant's hand-span. Ayla and colleagues interpreted these findings as evidence against the biomechanical account. In our opinion, the observation of relatively constant JNDs for mid-range-sized objects may in principle, however, be compatible with the biomechanical account. For this range of objects, the hypothesis predicts that the variability-boosting effect of Weber's law and the variability-dampening effect of biomechanical constraints with increasing object size (and vice versa for decreasing object size) might cancel each other out. For example, Weber's law would predict that the MGA variability should be smaller for the 20% object than the 60% object, whereas the biomechanical account would predict the opposite because the range of available hand openings is smaller for the 60% object than the 20% object. In summary, experiments on MGA variability in unimanual grasping do not seem to provide the most reliable way to test the biomechanical hypothesis. A more promising approach is thus to examine a form of grasping for which biomechanical limits are largely irrelevant for a wide range of object sizes: bimanual grasping.

This approach was adopted by Ganel et al. (2017). Bimanual grasping ensures the availability of a large range of safety margins and comfortable movements for a variety of object sizes as long as object sizes are considerably smaller than participants’ arm span. In the study, participants were asked to use two hands to estimate the sizes of objects or to grasp them. They employed a between-subjects design (*N* = 8 per condition) to examine the adherence of bimanual estimation and grasping to Weber's law in four conditions: (1) closed-loop bimanual grasping; (2) closed-loop bimanual estimation; (3) open-loop bimanual grasping; and (4) a closed-loop bimanual estimation task that controlled for the absence of haptic feedback by letting participants touch the object at the end of each estimation trial. They found that the responses in the perceptual tasks adhered to Weber's law, whereas the responses in the bimanual grasping task again violated Weber's law. Consequently, Ganel and colleagues (2017) argued that the violation of Weber's law in grasping cannot be attributed to biomechanical factors but instead supports the predictions of the PAM.

To our knowledge, the study by Ganel and colleagues (2017) has so far been the only study to investigate Weber's law in bimanual grasping/estimation. Given the history of controversial findings of studies investigating the claims of the PAM (e.g., Franz et al., 2003; Haffenden et al., 2001), it is important to test whether the finding that bimanual grasping violates Weber's law can be replicated. This seems particularly critical as some methodological details of Ganel et al.'s study (2017) appear to be worth addressing (e.g., between-subject variations, small sample sizes of *N* = 8, mixing of closed-loop and open-loop vision conditions). Given those limitations, it seems pertinent to examine whether the effects replicate when sufficient statistical power is ensured. In addition, there is another specific aspect of Ganel et al.'s study (2017) which may have affected their chances of observing Weber's law in grasping and which we aimed to reassess: the variations of the hands’ start position depending on object size.

In most grasping studies, participants are asked to place their fingers “pinched” at a start position prior to each grasping movement. This procedure ensures a common baseline for measuring how the size of the target object affects the emerging hand-opening during the movement. As discussed above, the standard measure to assess the internal representation of object size by the visuomotor system is the maximum opening of the hand (MGA), usually achieved at about 75%–80% of the movement time when moving the hand toward the target object (Jeannerod, 1984; Smeets & Brenner, 1999). Studies on Weber's law determine the JNDs in grasping as the variability (most commonly computed as standard deviations) in the size of MGA (e.g., Bruno et al., 2016; Ganel et al., 2008; Jazi & Heath, 2014). However, some studies have also examined aperture size and its variability throughout the grasping movement (e.g., Ganel et al., 2014; Heath et al., 2011, 2012; Holmes et al., 2011). Interestingly, these studies found that the variability of the hand-opening values was time-dependent: JNDs increased with object sizes (thus adhering to Weber's law) during the early phase of the grasping movement but remained constant (thus violating Weber's law) during the latter phase of the movement (Heath et al., 2011, 2012; Holmes et al., 2011). Consequently, it was suggested that the early part of the grasping movement is under the influence of the perceptual (ventral) system while the latter part of grasping movement is under the control of the visuomotor (dorsal) system.

However, this reasoning was subsequently challenged by Foster and Franz (2013) who argued that the finding constitutes a mathematical artifact. Specifically, they showed that the obtained data pattern of size-dependent variability being restricted to the early phase of the movement (pre-MGA) can be explained without any recourse to Weber's law. Instead, for any temporal function where temporal noise can be expected, the standard deviation (SD) of the function's output values will be proportional to the value of the function's first derivative. Applied to grasping, this means that the SDs of the aperture will vary in proportion with the aperture velocity. The aperture velocity prior to MGA is driven by the intended maximum hand opening (i.e., the larger the intended opening, the higher the opening velocity), which in turn is driven by the perceived target size (the larger the target, the larger the required hand opening). Consequently, aperture variability (JND) increases for larger objects prior to MGA. Hence, the effect is not indicative of Weber's law and by extension will not be informative about the relative contribution of perceptual and visuomotor streams to the different phases of the grasping movement (see also Ganel et al., 2014 for empirical support of this argument). Importantly, this reasoning also identifies an easy way to circumvent the problem. By restricting the analysis of aperture variability to MGA this confound can be avoided. At the point where MGA is reached, aperture velocity is zero and, thus, its variability is no longer size-dependent (Foster & Franz, 2013).

However, Ganel et al. (2017) suggested a different solution in their studies which allowed them to analyze the existence of Weber's law across the whole movement while at the same time avoiding the confound of aperture velocity. To eliminate the correlation between object size and pre-MGA aperture velocity, they adjusted the starting positions of the hands to the target size. For example, when participants had to grasp a 150 mm wide object, they started with the fingertips placed at the opposite ends of a 50 mm wide object; and when grasping a 450 mm wide object, they started with holding a 350 mm wide object. That is, the start object was always exactly 100 mm shorter than the target object. This procedure meant that the amount of hand-opening required was constant and independent of the size of the target object. Thus, the unwanted correlation between target size, opening-velocity, and aperture variability should be eliminated (see Ganel et al., 2014 for a similar method in unimanual grasping). Yet, it is possible that it may have introduced a different confound.

The potential issue is that the task may now become uninformative in relation to the question of whether the perceptual and the visuomotor system use different estimates for visual size. Since the target object was always 100 mm larger than the object held prior to movement onset, participants may complete the task successfully by always applying the same motor response, namely pushing their two hands apart by 100 mm. In this case, participants may no longer rely on visual estimates of size information in order to grasp the target successfully (in fact, after sufficient training they should be able to perform the task blindly) meaning that Weber's law would no longer be expected to hold true. If the tasks do not require accurate visual size estimates, then it is unclear what the performance-dissociations tell us about perception and action processing.

To conclude, the main premise of Ganel et al.'s (2017) study is convincing. If biomechanical constraints explain why Weber's law is violated during grasping, removing those constraints by using a bimanual grasping task should allow Weber's law to emerge. Thus, bimanual grasping seems to provide a valid test for the biomechanical hypothesis. However, by varying the hands’ start positions, they may have employed a task that did not necessarily require the estimation of visual size.

Here, we aimed to address this issue by testing directly whether effects differ depending on the hands’ start positions. For each task type (i.e., bimanual estimation and grasping), we tested a condition where the start position remained fixed (as for most previous unimanual grasping paradigms) and a condition where the start position was adapted and varied with the size of the stimulus (like Ganel et al., 2017). By using variability of MGA as an indicator of adherence to Weber's law, the confound between aperture velocity and object size is accounted for in the fixed start position condition (as aperture velocity is zero when MGA is reached). Importantly, we also employed a more powerful within-subject design and a larger sample size of *N* = 20 (see Methods section for more information on sample size choice). We also tested all conditions visually open-loop as it has been argued that online corrections in visually guided grasping can potentially mask the effects of Weber's law (Bruno et al., 2016). That is, grasping responses may be less variable than perceptual responses because participants can use visual feedback to correct and update their responses online during the grasping task but not during the perceptual task (e.g., Franz et al., 2009; Schenk et al., 2011; Utz et al., 2018) when performed with full vision (closed-loop). The advantage of open-loop conditions is that, according to the assumptions of the PAM, actions still rely on dorsal stream processing while at the same time the effect of online visual feedback is controlled for (see Schenk & Hesse, 2018 for review). Note that, according to the PAM, object visibility during the reaction time interval determines whether real-time computations of the dorsal or the stored ventral stream information are used to guide the movement (e.g., Goodale et al., 2004, 2005; Westwood et al., 2003). As long as vision of the target object is available during the movement initiation period, actions are supposed to rely on the dorsal stream. To date, most researchers agree that only open-loop grasping paradigms can provide conclusive evidence in support of potential perception-action dissociations (Bruno et al., 2016; Hesse & Franz, 2009; Schenk & Hesse, 2018).

Finally, the objects (i.e., polystyrene foam rods) used by Ganel et al. (2017, Figure 1) required relatively unnatural actions that artificially forced participants to grasp objects bimanually which would commonly be grasped with a (central) unimanual full-hand grip. In fact, Derzsi and Volcic (2020) reported that they found Weber's law-like pattern in the variability of the selected grasp positions (that rely on the object's perceived center of mass and hence are also assumed to require a size estimate) when similar rods were grasped with a more natural unimanual central grip. Hence, we opted for stimuli that would be naturally grasped bimanually in our study.

**Figure 1.**
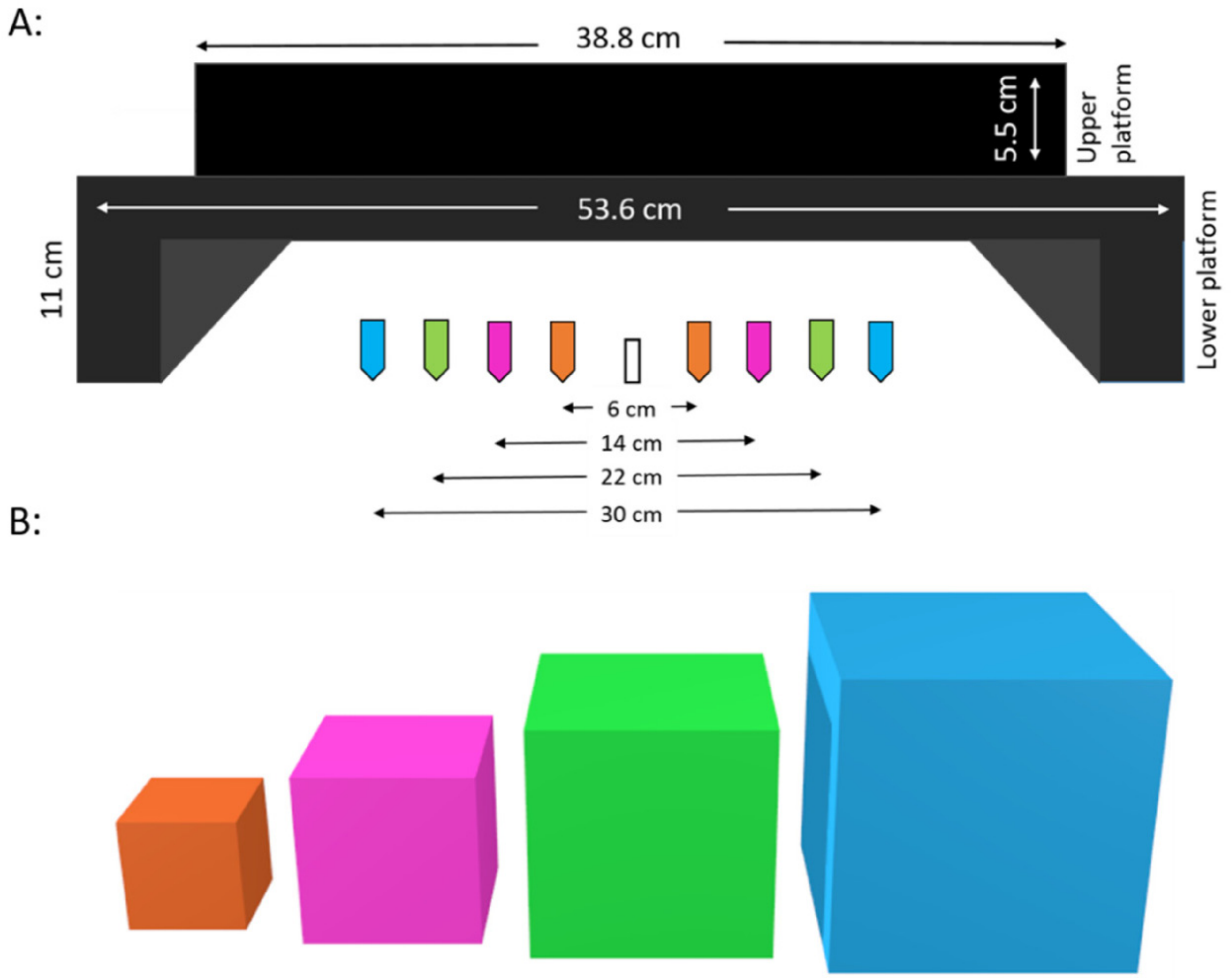
(A) Platform on which stimuli were placed during bimanual grasping and bimanual estimation with an illustration of the different start positions of the hands. (B) Schematic depiction of the stimuli used in the study. Note that colors are for illustrative purposes only to match the coloring of the start positions and clarify where hands were placed prior to either grasping or perceptually estimating a box of a certain size in the adapting start position condition. In the fixed start position condition, participants always started from the innermost orange markers for all box sizes. The boxes in the experiment were all uniformly black.

## Methods

### Participants

Twenty-two neurologically healthy volunteers with normal or corrected to normal visual acuity participated in the experiment. The data from two participants was excluded from analysis as they did not follow the instructions (i.e., placing their hands at the respective start positions and grasping the boxes on their sides). The remaining 20 participants (15 female) were between 18 and 37 years old and right-handed by self-report. The sample size of *N* = 20 was based on a G*Power 3.1 analysis (Faul et al., 2009). Using a mixed design (with task, manual estimation vs. grasping, as between-subject factor) and object size as a within-subject factor with four levels, Ganel et al. (2017) observed a large effect of *f* = .70 (η_p_^2^ = .34) for the interaction between the linear components of bimanual grasping and perceptual estimation for the JNDs in their study. Based on this finding, we defined *Cohen's f* as a standard (but more conservative) strong effect of .40 in our power analysis. For a within-subjects design with 1–*β* of .99, an effect size *f* of .40 (η_p_^2^ = .14) and an α level of .05 a required sample size of *N* = 20 was identified to detect an interaction effect between task and object size.

Undergraduate students received course credits for their participation. The study was approved by the School of Psychology Ethics Committee (PEC/4379/2019/10) at the University of Aberdeen and all participants provided written informed consent prior to participating in the study.

### Materials and Procedure

A TrakStar™ electromagnetic motion tracker (Ascension Technology Corporation, NDI) with a sampling rate of 240 Hz was used to record participants’ hand movements. One marker was placed on the fingernail of participants’ index finger on each hand. It was kept in place with white-tac and medical tape, exposing the pad of the finger so that participants received normal haptic feedback. Small Velcro strips were attached at the base of the index finger and at the wrist to keep the wire from each marker out of the way during the trials. Participants wore liquid-crystal shutter glasses (PLATO Translucent Technologies, Toronto, Ontario; Milgram (1987)) to control vision occlusion. The experiment was programmed in Matlab (Mathworks, Natick, MA, USA).

Participants sat in a well-lit room at a table (height: 72 cm, width: 92 cm) upon which two platforms were affixed (see Figure 1). The lower platform contained the TrakStar™ transmitter cube. The upper platform, on which the target boxes were placed, had a release button embedded. The target boxes were four hollow polystyrene cubes covered in black adhesive foil with side lengths: 160, 240, 320, and 400 mm (weights: 300, 400, 500, and 600 g). A wooden block was secured inside each cube to add some weight to the boxes. Participants adjusted the position of their chairs so that they could comfortably reach and grasp the largest target object.

**Figure 2.**
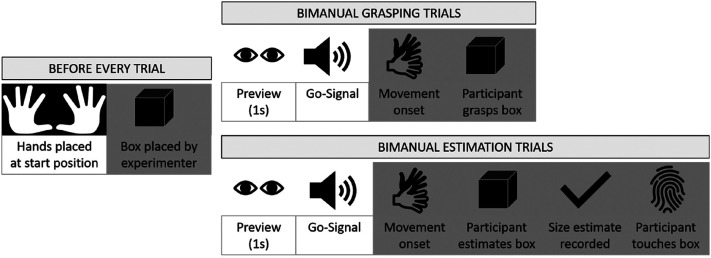
Timelines for grasping and estimation trials. Trial occurrences depicted on a white background were completed with full vision while trial occurrences depicted on a gray background were completed while vision was occluded.

Participants performed two different tasks: bimanual grasping (action task) and bimanual estimation (perceptual task). Figure 2 shows the timeline of the grasping and bimanual estimation trials. Before every trial, the shutter glasses opened so that the participants could place their fingers at the correct start position as instructed by the experimenter. The experimenter then closed the shutter glasses so that the participants’ vision was occluded while the box was placed in front of them on the upper platform. For the grasping trials, the box was placed on the release button in line with the front edge of the upper platform. Lifting the box triggered a signal indicating that the box had been moved. For the bimanual estimation trials, the box was placed 13 cm further back on the platform just beyond the release button to give participants sufficient space to make their estimations.

Once the experimenter had placed the box in the correct position, the trial was started manually with a keypress. In response to the keypress, the shutter glasses opened for 1 s to give participants a preview of the target object. After this preview period, an auditory cue (1,000 Hz, 100 ms) signaled participants to start their movement and to either grasp the box or to estimate its size. As soon as either of the hand markers had moved more than 25 mm away from the start position in 3D space, the shutter glasses closed, and participants performed their grasp or size estimate without vision of the target object and their hands (open-loop condition). In the grasping trials, participants were instructed to lift the box upwards and then gently place it back. In the manual estimation trials, participants were asked to indicate the size of the box with their hands as accurately as possible. Participants verbally indicated to the experimenter when they were satisfied with their estimate and the experimenter recorded the hand positions (representing the estimate) with a keypress. Once the response was recorded, participants were asked to touch the sides of the box, so that they received the same haptic feedback as in the grasping task (Ganel et al., 2017).

For each of the two tasks (i.e., bimanual grasping and estimation), there were two different conditions varying where participants had to place their hands prior to the start of the trial. In the “fixed start position” conditions, participants always placed their hands at the most central markers 6 cm apart (i.e., indicated in orange in Figure 1A). That means that in this condition, the distance that the hands had to move to reach the edges of the boxes in grasping, or to indicate their size during estimation trials, increased with the box size. In contrast, in the “adapting start position,” participants adjusted the start position of their hands prior to each trial depending on the box size. That means that in this condition, the distance that the hands had to move to reach the edges of the boxes in grasping, or to indicate their size during estimation trials, always remained constant: 50 mm for each hand independent of box size (see Figure 1A). The adapting start position conditions were designed in accordance with the procedures employed by Ganel et al. (2017). Start position markers were color-coded on the table-top (see Figure 1A). A third TrakStar™ marker was stuck to the table-top, aligned with the middle of the target object. The central markers were placed 60 mm apart (30 mm on either side of the midline to which the target object was aligned). Each subsequent set of markers was placed an additional 40 mm on either side further from the midline (as the side length of the cubes increased by 80 mm). The innermost markers (orange markers in Figure 1A) were used for all the trials in the “fixed start position” conditions, and for the 160 mm box in the “adapting start position” conditions.

Once the electromagnetic markers had been attached to the participants’ fingers, we took one calibration measurement with participants’ index fingers placed on the central markers. This allowed us to determine the baseline position of the fingers—a reference needed to determine the onset of the movement at all start positions which we used in turn to trigger the closing of the shutter glasses.

Both start conditions and tasks were blocked and partially counterbalanced across participants: half of the participants started with a grasping task and half started with a bimanual estimation task, half of the participants started with a central start position condition and the other half with a side start position condition. Participants never completed two successive grasping blocks or two successive bimanual estimation blocks—the task type always changed at the start of a new block.

Each box was presented 10 times within each block, resulting in a total of 40 trials per block and 160 in total. Within each block, the box sizes were pseudo-randomized. Each block was preceded by a minimum of four practice trials (one for each box) until the participant felt comfortable with the task. The duration of the experiment was ∼2 h including breaks between blocks.

### Data Analysis

The 3D position data of the two markers were filtered offline using a second-order Butterworth filter with a cut-off frequency of 15 Hz. Resultant velocity was calculated from the filtered 3D position data of the markers. Movement onset was defined as the first frame where the resultant velocity of one of the markers exceeded 0.05 m/s. The end of the grasping movement corresponded to the moment the box was lifted off the release button. Grasping trials in which the start or end of the movement could not be reliably determined (e.g., participants moved before the go-signal) were excluded from further analysis. This was the case for 16 out of 1,600 trials across all participants.

MGA was calculated as the maximum 3D Euclidean distance between the hands reached during the grasping movement. The hand aperture in the bimanual estimation trials was determined as the 3D Euclidean distance between the hands at the time of the keypress. Estimation trials in which participants moved their hands at the moment the aperture was recorded were excluded from further analysis. This was the case for four out of 1,600 trials across all participants. In line with previous literature (e.g., Ganel et al., 2008, 2017), JND values were defined as the SDs of the MGAs for each participant in each condition (box width, task type, start position combinations).^1^ Both MGA and JND data were initially analyzed using a 2 (*task type:* grasping vs. estimation) × 2 (*start position:* fixed vs. adapting) × 4 (*box width:* 160, 240, 320, and 400 mm) repeated-measures ANOVA.

We also calculated the endpoint variability of the contact points of the hands on the targets in 2D in the grasping conditions. To do so, we determined the position of each hand in depth (*y*-direction, sagittal axis) and height (*z*-direction, coronal axis) at the end of the grasping movement. 2D endpoint variability was computed by fitting a 95% confidence ellipse around *y* and *z* data points for each box size, start position, and hand (left-hand contact point and right-hand contact point) across the 10 repetitions. The surface areas of those ellipses (in mm^2^) provide a measure of the 2D endpoint variability (Hesse et al., 2016; Messier & Kalaska, 1997, 1999). Endpoint variability data was initially analyzed using a 2 (*hand:* left vs. right) × 2 (*start position:* fixed vs. adapting) × 4 (*box width:* 160, 240, 320, and 400 mm) repeated-measures ANOVA.

Whenever the assumption of sphericity was violated, Greenhouse-Geisser corrections were applied to *p*-values but full degrees of freedom are reported. Post-hoc comparisons were carried out using Bonferroni corrections for multiple comparisons. Values are presented as means ± 1 standard error of the mean (between subjects).

## Results

### MGA and JNDs

**Figure 3.**
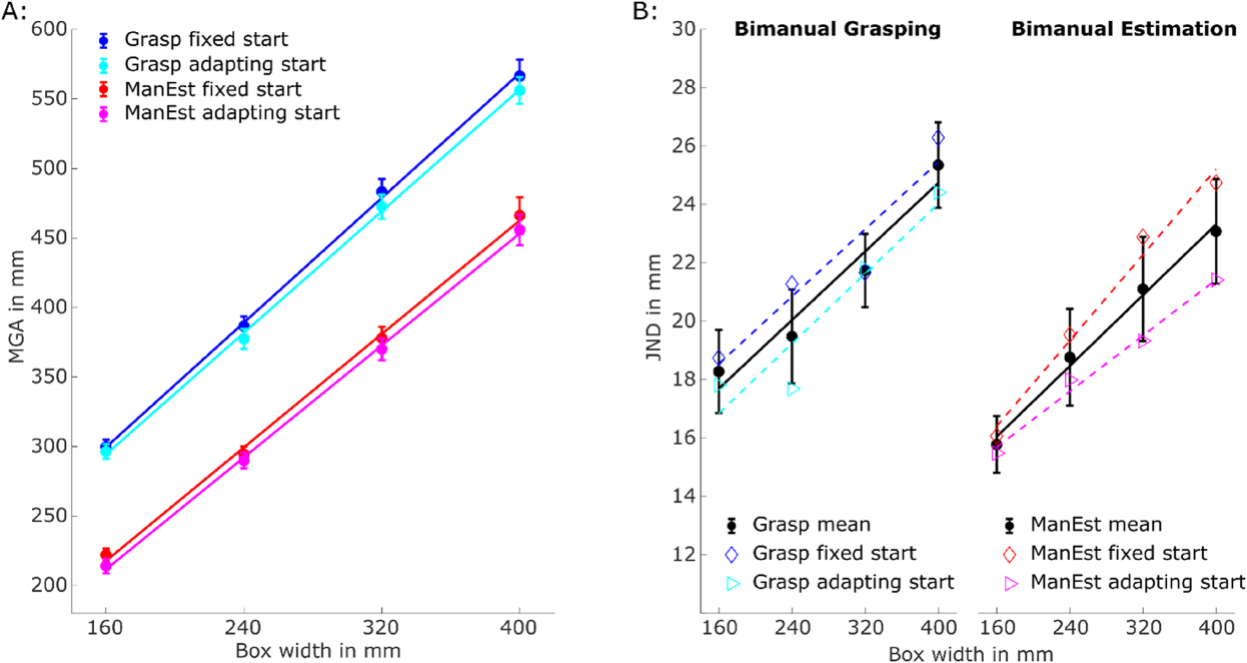
(A) MGA as a function of box width, task and start position. (B) JNDs for grasping and estimation tasks for all box widths averaged across start positions (black dots) and separate for the fixed (diamond symbols) and adapting (rightwards pointing triangle) start positions. All lines indicate linear regression lines (least square fit) to the data points. Error bars represent  ± 1 standard error of the mean (between subjects) in all graphs.

Abbreviations: MGA = maximum grip aperture; JNDs = just noticeable differences.

Figure 3A shows the mean MGAs for each box width in each condition. As expected, MGAs increased linearly with box width in all conditions. The 2 (*task*: grasping vs. bimanual estimation) × 2 (*start position*: fixed vs. adapting) × 4 (*box width*: 160, 240, 320, and 400 mm) repeated-measures ANOVA revealed a significant main effect of *task*: *F*(1,19) = 165.78, *p* < .001, η_p_^2^ = .90. On average, participants opened their hands about 93.5 ± 7.3 mm larger during grasping than during bimanual estimation. This effect reflects the fact that participants needed to open their hands wider than the width of the box to grasp it successfully, whereas bimanual estimation required participants to indicate box width as accurately as possible (i.e., no safety margin required). There was also a significant main effect of *box width*: *F*(3, 57) = 1263.89, *p* < .001, η_p_^2^ = .99, confirming that participants opened their hands wider for larger boxes (*p* < .001 for all pairwise comparisons). The main effect of start position was also significant, *F*(1,19) = 5.41, *p* = .031, η_p_^2^ = .22, with participants opening their hands slightly wider when starting their movements from the fixed central start position (8.1 ± 3.5 mm). Finally, there was a significant interaction effect between *task* and *box width*, *F*(3,57) = 7.12, *p* = .006, η_p_^2^ = .27. This interaction indicates that the increase in MGAs for the different box widths differed for grasping and bimanual estimation.

To determine the sensitivity of bimanual estimation and grasping to box width, linear regressions with the predictor *box width* and the dependent measure *MGA* were calculated separately for all conditions and participants. The slopes of these functions indicate the sensitivity to changes in box width. A 2 (*task*: bimanual estimation vs. grasping) × 2 *(start position*: fixed vs. adapting) repeated-measures ANOVA revealed that slopes were slightly steeper for grasping (1.11 ± 0.03) than for estimation (1.01 ± 0.04), *F*(1,19) = 7.48, *p* = .013, η_p_^2^ = .28. This mirrors the findings of Ganel et al. (2017) who also observed steeper slopes for bimanual grasping than for bimanual estimation. Neither the main effect of start position nor the interaction effect were significant (both *p* > .41).

Our main question was whether Weber's law, measured as the variability of MGAs, could be observed in grasping when biomechanical constraints were reduced. If Weber's law only holds for the perceptual system but not for the visuomotor system—as claimed by Ganel and colleagues (2008, 2017)—one would predict a statistically significant interaction effect between *task* and *box width*. In contrast, if perceptual and visuomotor tasks adhere to Weber's law, one would expect to find a main effect of *box width* and, more importantly, a linear increase in JND with increasing box width in both conditions.

JND data was initially analyzed equivalent to MGA using a 2 (*start position:* fixed vs. adapting) × 2 (*task*: grasping vs. bimanual estimation) × 4 (*box width*: 160, 240, 320, and 400 mm) repeated-measures ANOVA. This analysis revealed a main effect of *box width*: *F*(3,57) = 20.13, *p* < .001, η_p_^2^ = .51. There were no main effects of *start position* (*p* = .08, η_p_^2^ = .15) or *task* (*p* = *.*39, η_p_^2^ = .04) as well as no significant interaction effects between any of the factors (*all p* > .59). Most importantly, a linear contrast analysis of the means revealed a statistically significant effect of box width, *F*(1,19) = 50.01, *p* < .001, η_p_^2^ = .73, confirming that JNDs increased linearly with box width. Contrary to the findings of Ganel et al. (2017), we observed no significant interaction effect between the linear components of object width and task (*p* = .90*,* η_p_^2^ = .001), thus suggesting similar adherence of the MGAs to Weber's law in both bimanual estimation and grasping tasks (see Figure 3B).

To further investigate the linear increase in JNDs with box width in each task, we also fitted linear regressions for each participant in each task with the predictor box width and JNDs as the dependent measure (averaged across start positions). We then tested whether the slopes of these regression functions were different from zero using one-sample *t*-tests. For both grasping and estimation, these tests revealed that slopes were larger than zero (grasping: *t*(19) = 5.43, *p* < .001 and estimation *t*(19) = 5.10, *p* < .001). Furthermore, a paired-samples *t*-test indicated that the slopes were of similar size in both tasks, *t*(19) = 0.13, *p* = .90.

### Endpoint Variability

**Figure 4.**
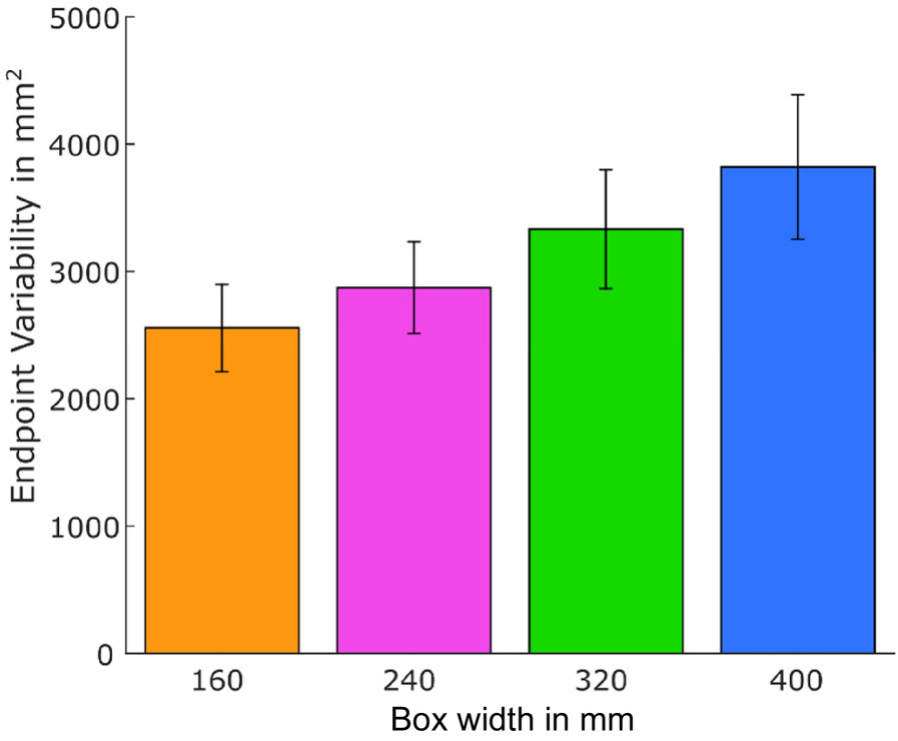
Endpoint variability for each box size aggregated across start position and hand. Error bars represent  ± 1 standard error of the mean (between subjects).

As the cubic nature of our stimuli meant that with increasing width of the target objects, the size of the contact surfaces increased too, one could argue that an increase in MGA variability (i.e., JNDs) may be attributed to the reduced accuracy required for grasping objects with larger contact surfaces. To test for this possibility, we determined the endpoint variability of the contact position in 2D space. A 2 (*hand:* left vs. right) × 2 (*start position*: fixed vs. adapting) × 4 (*box width*: 160, 240, 320, and 400 mm) repeated-measures ANOVA revealed a significant effect of box width on endpoint variability, *F*(3,57) = 4.66, *p* = .014, η_p_^2^ = .20 (see Figure 4). There were no main effects of hand (*p* = .11, η_p_^2^ = .13) or start position (*p* = .98, η_p_^2^ < .001) as well as no interactions between the factors (all *p* > .23). Thus, endpoint variability increased with increasing box size. As we currently do not know whether endpoint variability in grasping also increases/changes when stimuli of varying length but with constantly sized contact surfaces are grasped (as in Ganel et al., 2017), we also wanted to test if box width predicts MGA variability (i.e., JNDs) independent of the changes in endpoint variability.

To do so, we calculated a multiple linear regression with both endpoint variability and box size as independent variables and JND (i.e., SD of MGA) as the dependent variable. Preliminary tests confirmed that there was no multicollinearity between the two independent variables (*r*(80) = .24, Variance Inflation factor = 1.06), Durbin-Watson statistic indicated that values of the residuals were independent, as the obtained value was close to 2 (Durbin-Watson = 1.85), and no cases were biasing our model (all Cook's Distance values under 1).

The multiple linear regression analysis suggests that both variables, endpoint variability and box width, added statistically significantly to the prediction of JNDs, *F*(2,77) = 28.64, *p* < .001, *R*^2^ = .43. Evaluation of the β coefficients showed that both independent variables had a significant partial effect in the full model (endpoint variability: *t* = 6.13, *p* < .001, β = 0.54, box width: *t* = 2.85, *p* = .006, β = 0.25). Thus, Weber's law holds in grasping even if we account for the increase in endpoint variability for larger boxes.

## Discussion

The aim of this study was to investigate whether grasping (action) adheres to Weber's law when biomechanical constraints are reduced in a bimanual grasping task. Within the current debate on whether or not the absence of Weber's law in grasping can be considered as evidence for independent processing pathways for perception and action as proposed by the PAM (Milner & Goodale, 1995, 2006), it has been suggested that, alternatively, the violations of Weber's law in unimanual grasping may be due to biomechanical constraints in the maximum hand span when grasping larger objects (Schenk et al., 2017; Utz et al., 2015). Yet, a recent study by Ganel et al. (2017) that employed a bimanual grasping paradigm to account for potential biomechanical limitations in unimanual grasping suggested that even in situations where biomechanical constraints are largely absent, grasping still escapes Weber's law. Here, we revisited this question and were particularly interested in whether the failure to observe Weber's law in bimanual grasping may be attributed to an unconventional methodological element in the task employed by Ganel and colleagues (2017): the adjustment of the hands’ start position to object size prior to each trial.

To test this hypothesis, we investigated bimanual grasping (action) and estimation (perception) under two different start position conditions. In one condition, the fixed start position condition, participants placed both hands together centrally prior to movement onset. In the other condition (same as Ganel et al., 2017), we varied the hands’ start position depending on the size of the target object in such a way that the distance between the start position of the hands and the edges of the boxes remained constant for all object sizes. In contrast to Ganel et al.'s findings, we found that JNDs in bimanual grasping adhered to Weber's law independent of whether or not participants started from a fixed central start position or an adapting start position. This means that contrary to our expectation, variations in start position had no effect on the occurrence of Weber's law in grasping and manual size estimation. Crucially, the slopes of the regression lines fitted to the JNDs in estimation and grasping were very similar (i.e., 0.03 for both) and also of comparable size to other studies that observed adherence of visuomotor data to Weber's law (e.g., slope of 0.04 for pantomime grasping reported by Manzone et al. (2017)). Overall, our findings are thus in line with the suggestion that biomechanical constraints may have masked the effect of Weber's law in grasping during unimanual studies (Utz et al., 2015).

Given that the adjustment of the hands’ start position to object size prior to the action cannot explain the failure to detect Weber's law in grasping in Ganel et al.'s study, the question arises of why we found different results in our study. One obvious methodological difference between the two studies is the objects that were used as stimuli. Ganel et al. (2017) presented participants with elongated polystyrene rods which varied only in length while the area of the contact surface at the ends of the rods remained constant for all objects (Figure 1 in Ganel et al., 2017). In contrast, while our stimuli were lengthwise comparable to those used by Ganel and colleagues, their contact surfaces were much larger and, since our boxes were cubes, increased with the width of each box. Based on this, one could argue that reduced precision requirements and increased endpoint variability for larger contact surfaces could have caused the Weber-like behavior for the JNDs at MGA. However, a multiple linear regression analysis confirmed that the increase in JNDs with increasing box size in our study could not be solely attributed to variations in the required endpoint precision (i.e., endpoint variability) but that object width was a significant, and *independent*, predictor of JNDs. Thus, the required movement accuracy does not seem to account for the differences in findings.

Yet, one may wonder whether, if the required endpoint precision in grasping also contributes to the variability in MGA, the effect of object size may actually be overall smaller in grasping than in estimation. If this was the case, then what would our findings tell us about the processing of object size in perception and action? In our opinion, the fact that Weber's law can be observed, at least in certain grasping tasks, is clearly informative and interesting because it challenges the previous notion that Weber's law is violated in all (visually guided) action tasks. There are several factors that may explain the potential issue of variability increasing less with increases in target size in grasping than in perception. First and foremost, as discussed in detail by Schenk et al. (2017), even though MGA is commonly considered as a read-out of object size by the visuomotor system, it is not only determined by object size but also by a variety of other factors, such as the need to perform a safe, comfortable and efficient movement. (see also, Hesse et al., 2016). The relevance and contribution of those additional factors will depend on the specific task and stimuli (e.g., the position of the object relative to the position of the hand, object weight, object shape, etc.) and the reliability of MGA as an estimate of target size will change accordingly. Hence, we would not necessarily expect increases in MGA variability and increases in manual estimation variability for larger target sizes to be identical (as manual estimation provides a much purer measure for object size representations in perception than MGA for object size representations of the visuomotor system).

Another distinctive feature of our stimuli was that due to their size and shape, contact positions of the hands on the object were not directly visible to participants, but had to be inferred from the visible front side of the box. However, to our knowledge, there is no convincing theoretical reasoning within the predictions of the PAM for why visibility of contact positions prior to the grasp should affect how (and where) visual information is processed for perception and action. According to the PAM, the processing pathway is primarily determined by the purpose for which the information is used, that is, perception versus action—as long as visual information is available during movement programming (as is the case for visual open-loop conditions).

Ganel et al. (2008, 2017) claimed that the finding that JNDs increase linearly in both unimanual and bimanual estimation (perception) but not in unimanual and bimanual grasping (action), thus violating Weber's law, provides another line of support for the claim of the PAM that visual information is processed differently in the ventral and the dorsal streams. Here, we present evidence that JNDs adhere to Weber's law in a bimanual grasping task which clearly challenges the claim that findings on Weber's law in perception and action consistently dissociate and can therefore be considered as unequivocal evidence in favor of the PAM.

We would like to emphasize that our sample was more than double the size of the sample used in Ganel et al.'s study and that we used a within-subjects design, resulting in higher statistical power. Furthermore, we consistently employed visual open-loop conditions for all tasks, thus ensuring that differences between perception and action tasks cannot be based on the differential use of online visual feedback during grasping (Franz et al., 2009; Hesse & Franz, 2010; Utz et al., 2018). However, since at this point it is hard to determine whether the difference in findings between our study and the study by Ganel and colleagues (2017) could be attributed to some other, currently unknown, effects of the differences in our respective experimental procedures, we believe that only further replications will allow us to settle the question of whether or not Weber's law can reliably be observed in grasping tasks once biomechanical constraints that are present in unimanual grasping tasks are eliminated.

## Notes

1. Note that the pattern of results remains identical when inter-quartile range (IQR) is used as a more robust measure of MGA variability (see also Utz et al., 2018). Summary data for IQR is available at doi: 10.5281/zenodo.5115810.
